# EGFR-Signaling and Autophagy: How they Fit in the Cancer Landscape

**Published:** 2016

**Authors:** Amar B Singh

**Affiliations:** Department of Biochemistry and Molecular Biology, Fred and Pamela Buffett Cancer Center, University of Nebraska Medical Center and VA Nebraska-Western Iowa Health Care System, Omaha, NE, USA

**Keywords:** EGFR, Autophagy, Carcinogenesis, Metabolic stress, Treatment resistance

## Abstract

Key significance of an overachieving Epidermal Growth Factor Receptor (EGFR)-signaling in cancer aggressiveness and poor prognosis is well recognized. In accordance, EGFR is either amplified or mutated in majority of the cancers of epithelial origin, and therefore has been recognized as a principal target for anticancer therapy. However, despite initial clinical efficacy of the anti-EGFR therapy in cancer treatment, long-term attempt to mute the cancer boosting effects of EGFR-dependent signaling meets resistance in cancer cells. Notably, effects of EGFR activation are pleotropic. Also, under conditions of anti-EGFR therapy in cancer cells, feedback activation of the pro-survival signaling by activation of other growth factor receptors can occur. However, a critical role of autophagy in the resistance against anti-EGFR therapy is fast emerging. Interestingly, EGFR regulates autophagy in a context-dependent manner. Furthermore, EGFR deregulated tumors demonstrate differential dependence upon autophagy for their survival and growth. Also, inhibiting EGFR-signaling promotes autophagy. These intriguing considerations are complicated further by findings that EGFR regulates autophagy in kinase-dependent or independent manner. Thus, for effective clinical cancer treatment using anti-EGFR regimen, it is critical that we understand molecular details of the nexus between the EGFR-signaling and autophagy.

## Introduction

The epidermal growth factor receptor (EGFR) is a widely-studied and versatile signal transducer and has been highly conserved during the evolution. The EGFR-dependent signaling regulates key processes of the cell biology, including proliferation, survival, and epithelial differentiation during development and tissue homeostasis (reviewed in Ref. [1,2]). Notably, since the discovery of EGF in 1960’s and its receptor in 1980’s [3–5], our understanding of the EGF/EGFR signaling pathway has advanced significantly. Remarkably, these studies also demonstrated that aberrant EGFR signaling is a major characteristic of many human malignancies including colorectal, lung, breast, and head and neck cancer [6,7]. Of note, the well-established traditional function of the EGFR is to transmit extracellular mitogenic signals, in ligand-dependent manner, by activating multiple downstream signaling cascades including phospholipase C-gamma, Ras, and phosphatidylinositol-3 kinase (PI-3K) [8]. Traditionally, the common outcomes of the EGFR-mediated signaling in cancer cells are uncontrolled tumor proliferation and enhanced survival of the tumor cells. Yet another way, EGFR expression can modulate gene promoters and transcriptional regulators is by direct shuttling (of the receptor itself) from cell-surface to the cell nucleus [9]. However, resistance to the anti-EGFR therapy in variety of cancer types have suggested ingenious utilization of the potential antistress mechanism/s by cancer cells to overcome effects of the anti-EGFR therapies for inhibiting cancer cell survival and growth. Notably, the key significance of autophagy in maintaining cellular homeostasis and stress management is now well recognized (reviewed in Ref. [10,11]). In accordance, recent success using a combinational therapeutic approach inhibiting both, the EGFR-signaling and autophagy, has highlighted the nexus between the EGFR signaling and autophagy in regulating cancer cell survival and welfare [12,13]. However, EGFR induces and suppresses autophagy in cancer cells, and inhibiting EGFR-signaling also promotes autophagy [14–16]. Therefore, it is essential that we understand molecular regulation of the interplay between EGFR-signaling and autophagy in the regulation of carcinogenesis.

## EGFR and Carcinogenesis

The ErbB family, consisting of ErbB1 (EGFR), ErbB2 (HER2), ErbB3 (HER3) and ErbB4 (HER4), of proteins and their ligands form a complex system in which interactions occurring between the receptors and respective ligands affect the type and duration of the intracellular signals that derive from receptor activation [1,8]. Of interest, proteins of the ErbB family form either homo- or hetero-dimers following ligand binding, each dimer showing different affinity for ligands and different signaling properties [1,8]. Notably, the growth and development of cancer cells is thought to occur through multiple genetic events that cause fundamental changes in the pathways regulating cell differentiation, proliferation, survival and mobility. However, elevated level of the EGFR and/or its cognate ligands have been identified as common components of multiple cancer types, and appear to contribute to the transformation of cellular phenotypes, growth and survival of the tumor cells (reviewed in Ref. [17]). In this regard, a prior analysis using more than 200 published studies that had examined relapse-free-interval or survival data directly in relation to EGFR levels in over 20,000 patients found elevated EGFR levels in cancer cells of clinical prognostic value. This prognostic value was then considered significant in head and neck, ovarian, cervical, bladder and esophageal cancers (related to the overall survival rate of 70%) and of moderate prognostic value, correlating to poor survival rates in 52%, in gastric, breast, endometrial and colorectal cancer patients (reviewed in Ref. [18]). These findings although were of limited clinical relevance due to their sole reliability upon total EGFR levels and without any consideration of the receptor activation, did highlight the clinical significance of the EGFR expression in evaluating cancer risk assessment and clinical management. However, continued investigations in this area have now demonstrated that in spite of the receptor overexpression and/or activation in cancer cells and efficient inhibition of EGFR-signaling using anti-EGFR monoclonal antibodies and/or Tyrosine kinase inhibitors (TKIs) resistance to anti-EGFR therapy may occur. Thus, understanding the molecular mechanisms affecting cancer cell sensitivity or resistance to anti-EGFR inhibitors may be of help in deciding on treatment options, and in new translational studies.

## Autophagy, Cell Death and Cancer Progression

The normal cellular homeostasis is dependent upon balance between the biosynthesis and catabolism of macromolecules. Perturbation of this balance is an important event in facilitating growth of the cancer cells, and their adaptation to the changes in local or systemic nutritional environment (reviewed in Ref. [19,20]). Notably, under the conditions of nutrient deprivation, autophagy is the crucial biological process for organism survival as it participates in the maintenance of cellular homeostasis by controlling the quality of cellular proteins and cytoplasmic organelles. The term autophagy (“self-eating”) was introduced by Christian De Duve in the decade of the sixties, based on the observation by transmission electron microscopy, of double membrane vacuoles containing cytoplasmic material [21]. Nowadays, autophagy is defined as a cellular pathway by which cytoplasmic macromolecules and organelles are delivered to the lysosomes for degradation [22]. However, recent advances in this field have demonstrated that molecular control of autophagy is complex and outcome (cell survival versus cell death) may vary depending upon the cellular context. The involvement of the proteins with tumor suppressor and/or oncogenic properties at different steps of the pathway further implies that the role of autophagy must be considered in tumor progression and therapy considerations. Notably, autophagy can be involved in elimination of the cancer cells by triggering a non-apoptotic cell death program, suggesting its negative role in tumor development. In agreement with this tumor suppressor hypothesis, the generation of knockout mice for specific genes involved in autophagy (ATGs) has shown that defects in specific regulators of this process are associated with the development of a tumorigenic phenotype [23]. In contrast, as a stress response mechanism autophagy will protect cancer cells from low nutrient supply or therapeutic insults. This stress adaptation in cancer cells by autophagy is now believed to be crucial in cancer progression. In accordance, early induction of autophagy has been documented in cancer cells subjected to anti- cancer treatment targeting growth-promoting signaling mechanisms including EGFR-signaling. This is why, co-targeting EGFR-signaling and autophagy is now emerging as a prudent approach in inhibiting cancer cell growth compared to the targeting of only one axis (Table 1).

## EGFR, Autophagy and Causal Association

Traditionally, ligand-dependent induction of the kinase activity has been considered the principal method of an EGFR-dependent regulation of the cellular processes. However, independent of the kinase activity or ligand activation, EGFR can mediate cellular processes through its ability to physically interact with other proteins. In agreement with these reports, Ewald et al. showed that the kinase-dead EGFR K721R mutant retained the ability to confer survival against serum starvation-induced cell death despite the loss of its ability to respond to EGF or to stimulate cell growth [24]. This notion was further corroborated by the finding that survival of cancer cells is maintained by EGFR independent of its kinase activity [16]. In this study, authors went on to show that the kinase-independent EGFR prevents autophagic cell death by maintaining the intracellular glucose level through its interaction with and stabilization of the sodium/glucose cotransporter-1 (SGLT1) [16]. The recent study by Tan et al. further supports a kinase-independent role of EGFR in promoting autophagy as it demonstrated that kinase-inactive EGFR interacts with the oncoprotein LAPTM4B that is required for the endosomal accumulation of EGFR upon serum starvation. The kinase-inactive EGFR and LAPTM4B stabilize each other at the endosomes and recruit the exocyst sub complex containing Sec5. It was further shown that the kinase-inactive EGFR, LAPTM4B, and the Sec5 sub complexes are required for the basal and starvation-induced autophagy. Moreover, it was demonstrated that the LAPTM4B and Sec5 promote EGFR association with autophagy inhibitor Rubicon, which in turn disassociates Beclin-1 from Rubicon to initiate autophagy [14]. However, EGFR activation also suppresses autophagy. In this regard, recent study by Wei et al. reported role of the kinase- active EGFR in regulating autophagy. Here, authors showed that the kinase-active EGFR binds autophagy regulatory protein Beclin-1, leading to its multisite tyrosine phosphorylation and decreased Beclin-1 associated VPS34 kinase activity. EGFR tyrosine kinase inhibitor (TKI) therapy disrupted Beclin-1 tyrosine phosphorylation and binding to its inhibitors and restored autophagy in non-small-cell lung carcinoma (NSCLC) cells harboring a TKI-sensitive EGFR mutation. Furthermore, in NSCLC tumor xenografts, the expression of a tyrosine phosphomimetic Beclin-1 mutant led to reduced autophagy, enhanced tumor growth and resistance to TKI therapy. Thereby, authors concluded that the oncogenic receptor tyrosine kinases directly regulate the core autophagy machinery, which may contribute to tumor progression and chemoresistance [15]. Taken together, the causal association between the EGFR expression/mutation/signaling and autophagy is undisputable however research findings have clearly demonstrated that this causal association is complex, context-dependent and may produce contrasting outcome.

## Cancer Resistance to anti-EGFR Therapy and Autophagy

The EGFR amplification or expression of the EGFR variant 3 (EGFRviii) is generally associated with resistance to the conventional anti-cancer therapy, through potential activation of the pro-survival signaling and DNA-repair mechanisms. This is why, EGFR targeting was conceived to be a successful strategy to increase anti-cancer treatment efficacy. Nevertheless, these targeting strategies have only been proven effective in a limited percentage of human tumors. Recent knowledge indicates that EGFR deregulated tumors display critical dependence on autophagy to increase resistance to EGFR- targeting drugs [25]. In this regard, EGFR mutated lung adenocarcinoma cell lines, HCC827 and HCC4006, contained a subpopulation of cells that have undergone epithelial-to-mesenchymal transition and survived independent of the activated EGFR. Of note, these EGFR-independent cancer cells demonstrated higher levels of basal autophagy than their parental cells and thrived under hypoxic, reduced-serum conditions *in vitro* [13]. Most notably, depletion of the essential autophagy gene ATG5 by small interfering RNA (siRNA) or use of the chloroquine, an autophagy inhibitor, markedly reduced cell viability in these cells under hypoxic conditions and induced caspase activity [13]. Of interest, induction in autophagy is observed after targeting cancer cells with TKIs (Tyrosine kinase inhibitors) or cetuximab [13,25].

**Figure 1:**
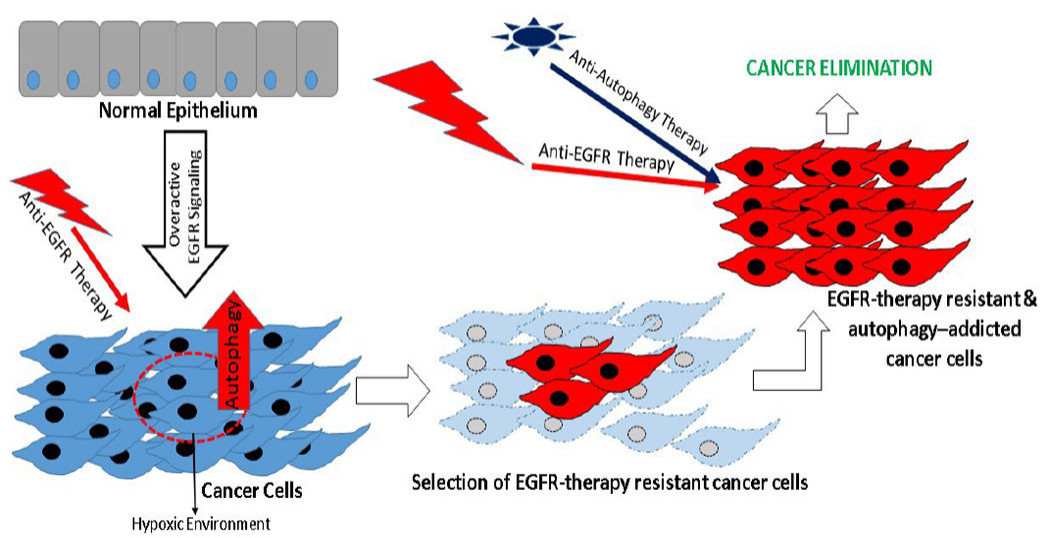
Cartoon depicting the role of the EGF receptor signaling in inducing cancer cell characteristics. Specific cells of the tumor mass, possibly due to the stress/nutritional challenge (like hypoxic environment), induce autophagy to overcome stress. Stress adaptive ability of these cells renders growth and survival advantages and resistance to anti-EGFR treatment. Therefore, combinational therapy using anti-autophagy treatment could boost the clinical efficacy of anti- EGFR therapy in treating cancer resistant to anti-EGFR regimen.

Interestingly, the effect of EGFR under normal condition seems to be mostly autophagy suppressive however becomes stimulatory during the metabolic stress. In accordance, in stably transduced glioblastoma cell lines and prostate cancer cells that express EGFRvIII, a faster and more pronounced autophagic response, during starvation or severe hypoxia, has been observed [13]. The enhanced autophagic response, in turn, seems to provide these cells with survival and growth advantages. The suppressive action of EGFR on autophagy activity and its opposing action during stressful conditions could result from signaling via different signal-transduction pathways. In this regard, potential interaction of EGFR with Rubicon and SGLT1 may play critical role. While, physical interaction of EGFR with Rubicon may help suppress inhibitory effect of the EGFR upon autophagy by restricting Baclin-1 phosphorylation, its partnering with SGLT1 may help improve energy availability through SGLT1-dependent glucose metabolism. Furthermore, the causal and contextual roles of the EGFR-PI3K-AKT-mTOR, EGFR-Ras and EGFR-Stat3 signaling, and mitochondrial homeostasis in this intricate interplay between EGFR and autophagy in regulating cancer progression would have to be considered. Also, conductance of such studies in context of the cancer stem cells, considered to be at the core of the cancer progression and resistance to therapy, would be pragmatic (Figure 1).

## Conclusion

The Epidermal growth factor receptor is one of the highly investigated molecules in the pursuit of cancer-centric research to understand molecular regulation of the cancer cell survival and plasticity. Encouraging outcome from these studies supported clinical efficacy of EGFR for anti-cancer therapy and led to the development of EGFR-targeting antibodies like cetuximab or panitumumab and TKIs like gefitinib, erlotinib, and lapatinib. However, expected success using these tools has eluded the scientist and clinicians equally due to the development of resistance by cancer cells to these therapies overtime. More recently, the potential of autophagy inhibition as therapy in cancer is being evaluated. Several reports indicate that cells and tumors with amplified or over activated EGFR are particularly sensitive to the autophagy inhibition for growth, survival, and resistance to conventional therapies. Additionally, resistance to EGFR-targeting therapies can also be reduced by autophagy inhibition. Inhibition of autophagy may therefore provide a novel treatment opportunity for EGFR-overexpressing tumors and should be pursued clinically.

## Figures and Tables

**Table 1:** A comprehensive list of the anti-ErbB family receptor therapies approved by the FDA for the clinical use to treat cancers.

Drug Name	Erbb Family Receptor Targeted	Cancer Type Treated
Gefitinib/Iressa	Tumors with EGFR exon 19 deletions or exon 21 (L858R) substitution mutations	First-line treatment of metastatic Non-small Cell Lung Cancer (NSCLC)
Erlotinib/Tarceva	An epidermal growth factor receptor (EGFR) inhibitor	Non-small Cell Lung Cancer or Pancreatic Cancer that has spread to other parts of the body (metastatic)
Lapatinib/Tarceva	Dual tyrosine kinase inhibitor which interrupts the HER2/neu and epidermal growth factor receptor (EGFR) pathways	HER-2 positive Breast Cancer
Cetuximab/Erbitux	EGFR receptor signaling	Colorectal Cancer, Head and Neck Cancer
Panitumumab/Vectibix	EGFR receptor signaling	Colorectal Cancer
Vandetanib/Caprelsa	Targets multiple kinase receptors including EGFR, VEGF, RET-tyrosine kinase	Thyroid Cancer
Necitumumab/Portrazza	EGFR receptor signaling	Metastatic Squamous Non-small-cell Lung Carcinoma (NSCLC)
Osimertinib/Togrisso	EGFR T790M mutation-positive metastatic non-small cell lung cancer	Metastatic Non-small Cell Lung Cancer
